# Using Google Street View for virtual observations of neighborhoods and dwelling units: A feasibility study

**DOI:** 10.1371/journal.pone.0307272

**Published:** 2024-08-01

**Authors:** Ting Yan, Xin (Rosalynn) Yang, Hanyu Sun, David Cantor

**Affiliations:** 1 Westat, Rockville, Maryland, United States of America; 2 Google, Washington, District of Columbia, United States of America; Sabancı Üniversitesi, TÜRKIYE

## Abstract

In face-to-face household surveys, field interviewers are sometimes asked to make notes of characteristics of the dwelling unit on the sampled address as well as its surroundings before making contact with a household member living at the sample address. Field interviewer observations of this kind are used to improve efficiency of field data collection and to be used as nonresponse adjustment. However, field interviewer observations can be expensive and the quality of observations needs to be improved. Recently, survey organizations start to utilize Google Street View to conduct virtual observations of the dwelling unit and the neighborhood. This paper reports a feasibility study that evaluates the feasibility of using virtual observations, assesses its agreement with field interviewer observation results, and examine whether virtual observations correlate with survey response status and survey estimates. We found moderate to high agreements between virtual and interviewer observation results. We also found that some observation results are significantly related to response status and survey estimates. However, virtual observations using GSV have coverage issues, which could limit their potential use.

## Introduction

### Background

In the era of declining survey response rates, survey researchers utilize data generated in the survey production process to facilitate data collection and to reduce survey errors [1]. In face-to-face household surveys, this usually includes information reported by address listers, observations conducted by field interviewers about neighborhoods and/or the dwelling units, and contact history data recorded in computer databases. Generated in the operational process and available for both respondents and nonrespondents [2], these auxiliary data can be used to monitor fieldwork progress [3], prioritize cases in the field [4], inform adaptive survey designs [5], and construct nonresponse adjustment weights [6].

Of interest to this paper are field interviewer observations of the sampled addresses and the neighborhood in which sampled addresses reside [6]. To conduct observations of this type, interviewers are instructed to walk around the sampled address, observe the characteristics of the sampled address, check the surroundings of the sampled address, and record their observations in an electronic system (e.g., via a coding form) prior to making any contact with any household member living at the sampled address. Commonly collected observation measures include unit type, access barriers, conditions of the unit, presence of children, and indicators of safety concerns [7–10].

Neighborhood and housing unit characteristics are important to survey research in general and crime and victimization surveys in particular. Research has shown that people living in urban cities [11], neighborhoods of higher crime rates [11], neighborhoods with lower income [12], neighborhoods with renters and/or apartment buildings [12] tend to have a lower propensity to respond to survey requests. In addition, these characteristics are also related to crime rates and victimization experiences [13]. As a result, neighborhood and housing unit characteristics are ideal candidates for adaptive designs and for nonresponse adjustment and are important information to collect as they are related to both response rates and survey estimates of crime and victimization [14].

Although interviewer observations are handy to collect, studies evaluating the quality of such observations found that they are potentially prone to measurement error ([10], see [2] for an exception), interviewer variance [6, 10], as well as varied missing data rates [9, 10, 15]. Evidence is mixed on the predictive power of interviewer observations on response propensity [10]. One study showed that interviewer observations of housing units have more predictive power of response status than area level information [16]. Two other studies only found limited ability of these observations to predict response status [9, 17, 18]. Another study assessing interviewer observations in the National Health Interview Survey found that although interviewers did record the observations, they did not necessarily conduct the observations prior to first personal contact as instructed [8].

With the development of remote sensing technologies used in geo-spatial data collection, Google Street View (GSV) has become a popular resource to virtually inspect locations searched via Google Map. First released in the United States in 2007, GSV provides interactive panoramic views of streets and environment to the public for free. GSV images are traditionally collected by cars equipped with special cameras to capture photos and the photos are then aligned and stitched together to offer a 360-degree view of an area. More recently, mobile phone users could also contribute to GSV by uploading live view pictures via the Street View app, which allows for more frequent updates and wider coverage of locations.

Past decades have seen an increasing number of studies using GSV in various disciplines, as noted in [19]. For example, GSV images have been used in urban planning studies to assess the abundance of street greenery and identify land-use information [20, 21]. In the public health literature, several studies have measured neighborhood walkability and facility access with virtual observations conducted with GSV and have examined such measures as correlates of health outcomes [22–24]. With GSV, virtual observations can be conducted remotely without going into the field, saving time and cost [19, 25]. Recently, advanced computing algorithms have been applied to GSV images [26]. However, past studies have noted potential pitfalls with the use of GSV for research purposes, including issues with coverage and low-resolution images [27, 28].

To carry out virtual observations, most studies so far used street segment or block face as units of observation and collected measures of street conditions and neighborhood disorder [21]. For example, Wu and colleagues developed a residential environment assessment tool that utilized GSV images to identify litter on the street level and broken windows on the property level in both urban and rural areas [29]. Odgers and colleagues evaluated street safety, decay, and disorder via GSV observations in the UK to assess children environment risk [30]. Studies checking agreement between GSV and field observations found higher concordance for items related to objective measures and lower for subjective items [31]. In addition, small and intermittent items (such as litter) were found to be difficult to identify via virtual observations [21, 27].

Two studies have evaluated virtual observations through GSV against field interviewer observations in household surveys. Vercruyssen and Loosveldt showed that virtual observations and interviewer observations were similar in predicting nonresponse [28]. Ren and colleagues evaluated both types of observations on completeness, validity, variability and/or reliability, and predictive power [10]. They found that interviewer observations have less missing data and are more predictive of response status than virtual observations [10]. But both types of observations need improvements on validity and reliability [10].

The current study extends the research on virtual observations through the use of GSV and attempts to address the following four research questions:

**RQ 1.** What’s the availability and quality of GSV images for conducting virtual observations?
**RQ 2.** How well do virtual observations conducted via GSV agree with field interviewer observations?
**RQ 3.** How accurate are virtual observations conducted via GSV compared with self-reported data?
**RQ 4.** Can virtual observations conducted via GSV predict screener response status and key survey outcomes in a crime survey?

To our knowledge, this study is the first one to quantify the availability and quality of GSV images and the first to examine the predictive power of virtual observations on key survey outcomes.

## Methods

This study used addresses sampled for the 2020 National Crime Victimization Survey Redesign Field Test (NCVS)-R. NCVS collects data on personal and household victimization with a nationally representative sample of residential addresses. Interviews were conducted using a Computer-Assisted Personal Interviewing (CAPI), with a small number being administered by phone from the interviewer’s home. Data collection on October 28, 2019, and continued through March 31, 2020. We obtained informed consent by having field interviewers reviewing the consent with each sampled person and asked for their verbal agreement. Field interviewers were allowed to start the survey only after obtaining the verbal agreement. There was no other documentation of verbal consent, and no witness was required to be present during the verbal consent process. We obtained parental permission to interview anyone under 18 before approaching the youth for an interview. Youth were also administered an assent, similar to that used for the adult consent, to get assent. Interviews were conducted on youth respondents after obtaining both parental consent and youth assent. NCVS-R was reviewed and approved by Westat Institutional Review Board.

For this study, addresses of 1,341 sampled dwelling units were randomly selected from the NCVS-R sample, consisting of a mix of screener respondents and nonrespondents, urban and rural addresses, as well as various self-reported crime and victimization status.

### Field interviewer observations

Field interviewers were instructed to conduct on-site observations of household and neighborhood conditions of sample addresses before contacting the sample address. Interviewer observations cover dwelling unit (DU) type, neighborhood income level, any signs of children, and street and neighborhood conditions. Observations are displayed in Table 1.

**Table 1:** List of virtual observational items.

Observations about dwelling unit (DU) through both GSV and by field interviewers
Dwelling unit type (1 unit; 2+ units; mobile home/trailer or recreational vehicle; some other type of residential structure; non-residential/not a DU)
Presence of children (yes; no)
Presence of any security measures (must pass through a fence or a barricade to enter; intercepted by doorkeeper, guard, or receptionist; intercom or phone needed to gain access; surveillance camera; saw dog on premises; sign on building indicating security service protection or burglar alarm; other warning signs; bars on windows or doors; other; none)
Signs dwelling unit not well kept (yes; no)
Observations about neighborhood through both GSV and by field interviewers
Neighborhood income level (low income; high or middle income)
Neighborhood safety level (definitely safe; fairly safe; unsure; fairly unsafe; definitely unsafe)
Signs neighborhood not well kept (yes; no)
Observations about neighborhood through GSV only
Street Condition (good; fair; poor; under construction)
Whether any unit in bad condition (yes; no)
Presence of graffiti (yes; no)
Availability and quality of street view images through GSV only
Whether Google Street View is available (yes; no)
Whether can confirm viewing the correct DU (yes; no)
Whether a close view available (yes; no)
Presence of inconsistency in images for street segment (yes; no)
Year/month image captured

### Virtual observations via GSV

Two virtual observers were asked to conduct virtual observations with GSV images. To retrieve street view images, virtual observers were instructed to first search for the sample address using Google Maps (maps.google.com) and browse the street view images available for the area. Virtual observers were instructed to confirm whether they were viewing the correct dwelling unit by checking the housing number visible on the housing structure. For condos and apartment buildings where the exact unit could not be directly observed, virtual observers were advised to observe building structure as a proxy for the dwelling unit. To evaluate neighborhood conditions, virtual observers were directed to browse street view images for adjacent blocks around the address. When GSV images were unavailable for a dwelling unit, virtual observers were asked to use Google Earth View to identify the land type and dwelling unit type and to indicate other observational items as missing.

Virtual observers conducted the same observations as field interviewers, as shown in Table 1. In addition, virtual observers were instructed to provide additional information on the quality of street view images, such as whether the images display any inconsistencies.

Two virtual observers each conducted virtual observations for half of the addresses and double coded a random selection of 50 addresses. An average of Cohen’s kappa coefficients is 0.61 based on the 50 double-coded addresses. The two virtual observers met with the research team and resolved all inconsistencies and applied the resolutions to the rest of addresses. After all addresses were coded, the team met and resolved all inconsistencies.

## Results

### Availability and quality of GSV images for virtual observations

**Fig 1:**
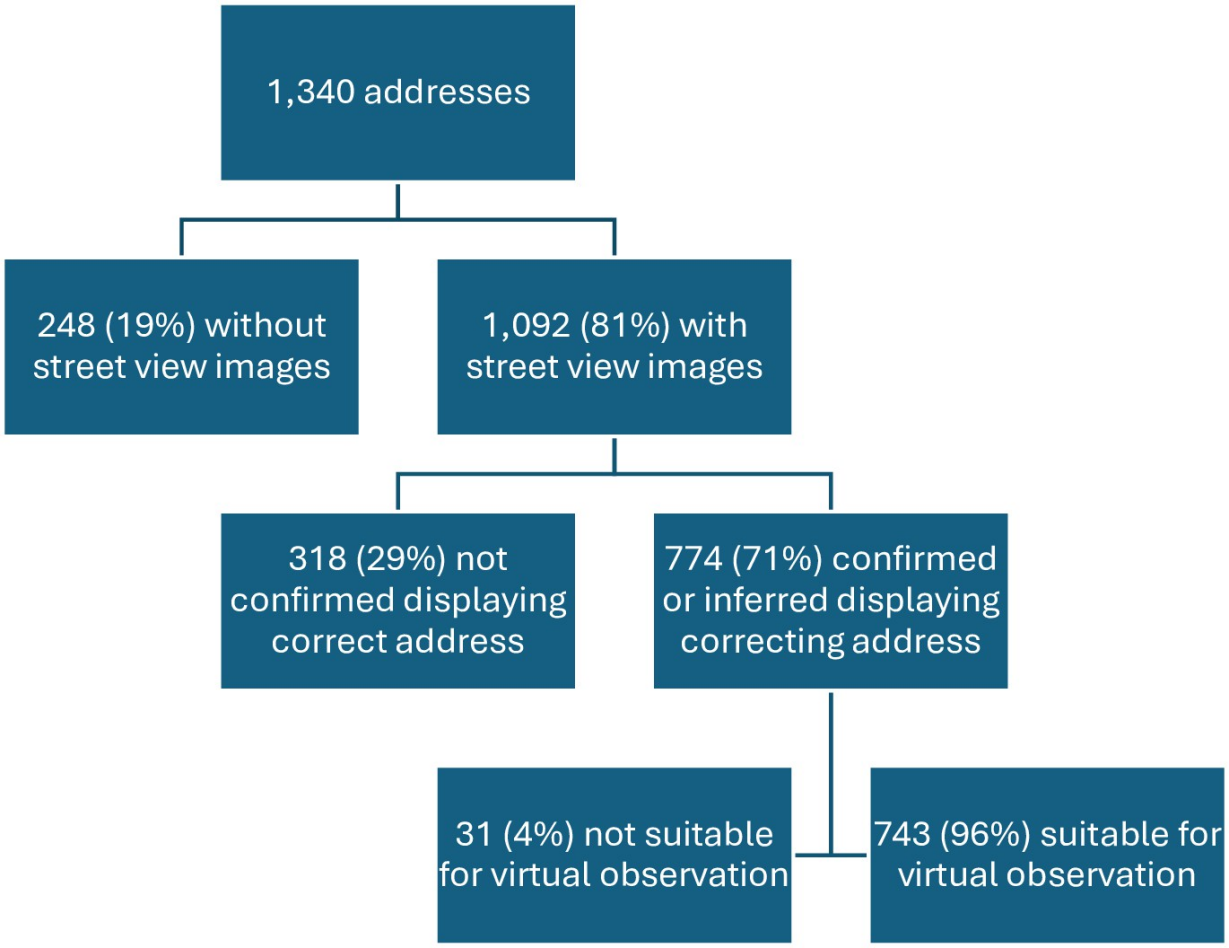
Availability and quality of street view images.

We first examined the availability and quality of GSV images of selected sample addresses. As shown in Fig 1, out of 1,340 sample addresses, about 19% do not have any street view images available. Among the 1,092 addresses with street view images, virtual observers could verify that 71% of these images display the correct dwelling units. This is done via confirming the housing number on the housing structure or in the area (e.g., visible on the sidewalk, printed on the postal mailbox in front of the unit) or by inferring the housing number by checking its adjacent housing units.

**Fig 2:**
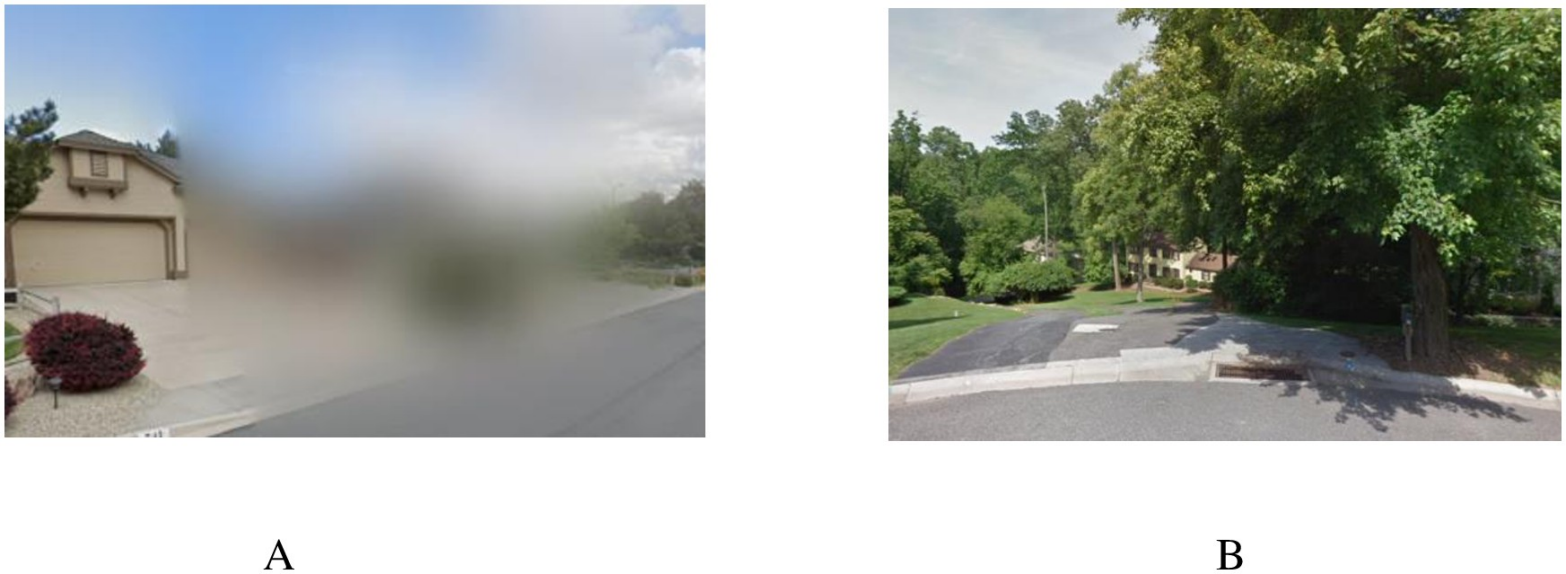
Examples of street view images not suitable for virtual observation. (A) Image is partially missing. (B) Housing unit is too far to observe in detail.

Among addresses that virtual observers were able to confirm or infer that the images are for the correct addresses, there are 31 addresses with street view images that are not suitable for observations. These images are partially missing or blurry (see an example image in Fig 2), too far to observe in detail (an example image is shown in Fig 2), or contain inconsistencies (e.g., having multiple images stitched together that affected virtual observation of the unit). After excluding the cases with aforementioned reasons, our analysis dataset contains 743 addresses with virtual observations conducted via good quality Google Street View images. In other words, virtual observations can only be conducted on 55% of sample addresses.

### Agreement between virtual observations and field interviewer observations

To answer Research Question 2, we assessed the agreement between the two sets of observations by computing the proportion of addresses with the same observations from virtual observers and field interviewers (*p*_*a*_). In addition, we calculated Cohen’s kappa as:

k=pa−pe1−pe

where *p*_*a*_ is the agreement rate and *p*_*e*_ is the agreement rate expected by chance.

As implied by the formula, Cohen’s kappa takes into consideration of chance agreement whereas the agreement rate (*p*_*a*_) does not.

Table 2 presents agreement rates and kappas based on 661 addresses that have both virtual observations and field interviewer observations data. “Dwelling unit type” exhibited the highest agreement rate among virtual observers and field interviewers; they agreed on 94% of the addresses with a Cohen’s kappa value of 0.87, consistent with earlier findings [10]. The percent of addresses coded as a one unit or single family house is 68% by virtual observers and 67% by field interviewers.

**Table 2:** Agreement between virtual and field observations (n = 611).

Observational item	Cohen’s kappa	Agreement rate	% of Addressed Coded as Yes or Modal Category^1^	
			Virtual Observations	Field Interviewer Observations
Dwelling unit type	0.87	94%	68%	67%
Signs dwelling unit not well kept	0.24	85%	8%	14%
Presence of any security measures^2^	0.41	84%	22%	10%
Signs neighborhood not well kept	0.16	84%	5%	15%
Presence of children	0.18	82%	12%	13%
Neighborhood income level	0.39	77%	18%	30%
Neighborhood safety level^3^	0.33	73%	74%	75%

Notes:

^1^The percentages are the percent of addresses coded as one unit or single family house, having signs that the dwelling unit was not well kept, having any security measures, having signs that the neighborhood was not well kept, having children at home, located in a low income neighborhood, and located in a safe neighborhood.

^2^Virtual observers and field interviewers can select more than one security measure on the observations form. A dummy coded measure (having at least one security measure vs. not having any security measure) is used for this table.

^3^This observation item has five response categories as shown in Table 1. For this table, we collapsed the two safe options and the two unsafe options and used three categories for agreement and kappa calculation.

Although other observational items only show fair to slight agreement according to Cohen’s kappa values [32], the agreement rates are all above 70%. The low kappas are likely the results of skewed distributions and/or the presence of empty cells [33, 34]. But it also points to the need to improve the observational items and improve the training of both the field interviewers and virtual observers.

Overall, field interviewers observed more addresses to have signs that the dwelling unit was not well kept, signs that the neighborhood was not well kept, and to be located in a low income neighborhood than virtual observers. Virtual observers judged more addresses to have at least one type of security measures with the aid of GSV images than field interviewers.

### Validating virtual observations against survey data

To address Research Question 3 on accuracy of virtual observations, we took advantage of two self-reported measures available in the survey data–the number of children in the household and household income–and use them to assess accuracy of virtual observations.

We first compared virtual observations of presence of children at a dwelling unit to self-reported number of children on 436 addresses that have both pieces of information available. The overall agreement rate between self-reports and virtual observations is 79% and kappa is 0.10. For comparison purpose, the agreement rate between self-reports and field interviewer observations is 78% and kappa is only 0.01. To gain a deeper understanding of the accuracy of virtual observations of presence of children at dwelling units, we further divided addresses into those self-reported having children (n = 57) and those self-reported *not* having children (n = 379). As shown in Table 3, among addresses that self-reported having children, 23% of the addresses were observed to have signs of children through virtual observations whereas field interviewers observed signs of children in 14% of them. By contrast, among addresses reported *not* having children, virtual observers and field interviewers correctly flagged 87% of them as having no signs of children. It seems that virtual observations through GSV are more accurate than interviewer observations in identifying households with children.

**Table 3:** Comparing observations to survey self-report data: Presence of children (n = 436).

Survey self-report	Virtual observations	Interviewer observations
% Observed signs of children among addresses self-reporting having children (n = 57)	23%	14%
% Observed *no* signs of children among addresses self-reporting *not* having children (n = 379)	87%	87%

Next, we used self-reported household income to evaluate virtual observers’ and field interviewers’ observation of neighborhood income level. Specifically, if the self-reported household income is less than $50,000, we code that the household lives in a low-income neighborhood. If the self-reported household income is $50,000 or more, then the household lives in a middle- or high-income area. We acknowledge that household income does not completely capture neighborhood income level even though the two are expected to be correlated.

The overall agreement rate between self-reports and virtual observations on neighborhood income is 61% with a kappa of 0.22 whereas the overall agreement rate between self-reports and field interviewer observations is 68% with a kappa of 0.36. Not surprisingly, the overall agreement rate between self-reported household income and observed neighborhood income is lower than that between self-reported presence of children in a household and observed presence of children at a dwelling unit.

Table 4 shows the comparisons of virtual and interviewer observations against determination through self-reported household income. Both virtual observers and field interviewers are better at characterizing neighborhood income level for addresses located in middle- or high-income neighborhoods. For addresses with a self-report household income equal to or higher than $50,000, virtual observers using GSV correctly judged 92% of them as located in a middle or high-income neighborhood and field interviewers correctly judged 86% of them as located in a middle or high-income area. Again, virtual observations are closer to respondents’ self-report than interviewer observations. However, the reverse is true for low-income neighborhoods. About one third of addresses with a self-reported household income less than $50,000 were correctly coded as located in a low-income neighborhood via virtual observations. By contrast, field interviewers correctly coded 51% of them in a low-income neighborhood.

**Table 4:** Comparing observations to survey self-report data: Neighborhood income level (n = 360).

Survey self-report	Virtual observations	Interviewer observations
% Observed as low-income neighborhood among addresses with self-reported income less than $50,000 (n = 181)	30%	51%
% Observed as middle or high-income neighborhood among addresses with self-reported income > = $50,000 (n = 179)	93%	86%

### Predicting screener response status with virtual observations

To assess the association between virtual observations and the address’ screener response status, we fit a logistic regression model to predict the likelihood of sample addresses responding to the screener survey using only virtual observations. As shown in Table 5, virtual observations of neighborhood income level and neighborhood safety are significantly correlated with sample addresses’ response status–households observed as located in a low income neighborhood and households observed as located in a fairly or definitely safe neighborhood are more likely to be screener respondents, consistent with literature [11, 12]. Virtual observations of dwelling unit type and signs of neighborhood not well kept are marginally predictive of households’ screener response status. The pseudo-R square of the model is 0.04.

**Table 5:** Logistic regressions predicting the likelihood of responding to survey screener using observations.

	Virtual Observations	Field Interviewer Observations
	Odds Ratio	Odds Ratio
Dwelling unit type: single unit (reference category = multiple units or mobile homes)	1.492^	1.762*
Presence of children	1.616	1.093
Presence of security measures	0.796	0.436*
Signs dwelling unit not well kept	0.973	1.128
Neighborhood income level: Low income (reference category = high or middle income)	2.162*	1.778*
Neighborhood safety level: Unsafe or unsure about safety (reference category = safe)	0.587*	0.495*
Signs neighborhood not well kept	0.477^	1.313
Pseudo-R Square	0.04	0.04
n	584	610

*p < .05,

^*p* < .10

For comparison purpose, we fit the same logistic regression model using field interviewer observations to predict sample addresses’ likelihood to participate in the screener interview. The pseudo-R square is also 0.04 but field interviewer observations of dwelling unit type, presence of any security measures, neighborhood income level, and neighborhood safety level are significant predictors of sample addresses’ screener response status.

### Predicting household level crime incidence with GSV observations

We also examined the associations between virtual observations and self-reported victimization experience at the household level. Three logistic regression models are fit with one each predicting whether the household had reported any personal crime, any property crime, or any type of crime (including either personal or property crime). The model estimates of virtual observations (in odds ratio), together with model estimates of field interviewer observations, are displayed in Table 6.

**Table 6:** Logistic regressions predicting likelihood of reporting crime incidence with observations.

	Virtual Observations			Field Interviewer Observations		
	Personal crime	Property crime	Any crime	Personal crime	Property crime	Any crime
Dwelling unit type: single unit (reference category = multiple units or mobile homes)	0.789	0.624^	0.563*	0.716	0.697	0.608*
Presence of children	1.732	1.415	1.382	1.002	0.749	0.953
Presence of security measures	1.215	1.087	1.052	0.464	2.269^	1.965
Signs dwelling unit not well kept	1.758	0.632	0.941	0.845	1.586	1.734^
Neighborhood income level: Low income (reference category = high or middle income)	0.778	1.759^	1.241	0.446^	1.32	1.015
Neighborhood safety level: Unsafe or unsure about safety (reference category = safe)	1.674	1.085	1.516	2.222^	1.039	1.123
Signs neighborhood not well kept	0.963	2.522	1.585	3.430*	0.978	1.372
Pseudo-R Square	0.02	0.05	0.04	0.05	0.05	0.05
n	405	405	405	423	423	405

*p < .05,

^*p* < .10

None of the virtual observations is significantly related to reporting of personal crime but field interviewer observations of signs neighborhood not well kept significantly increased the likelihood that the sample address would report personal crime victimization.

Two virtual observations (dwelling unit type and neighborhood income level) are marginally significant predictors of property crime whereas field interviewer observations of presence of children is marginally related to reporting of property crime victimization.

## Discussion

This study evaluated the use of Google Street View to conduct virtual observations of the sampled addresses in a household crime survey. We first examined the availability and quality of GSV images and found that 19% of the addresses did not have GSV images. Among those with GSV images, 71% of the addresses could be confirmed by coders that the images showed correct dwelling units. Another 4% of addresses confirmed as showing correct dwelling units are not suitable for virtual observations. As a result, virtual observations can only be conducted on 55% of sample address. In other words, close to half of the addresses were not covered if researchers only relied on virtual observations via GSV. These findings are consistent with literature on incomplete coverage of GSV [22].

We further checked the recency of the images and found that among the dwelling units that had street view images available, about half of the images were taken in the three years prior to virtual observations (2017, 2018, 2019). There were also 4% addresses with images captured 10 years ago, an empirical evidence for the time lag issues cited in [10, 22]. We advise that researchers interested in using virtual observations to note the time lag issues and to evaluate the impact of the time lag on the feasibility of using virtual observations and on the accuracy of the virtual observation results.

The agreement between interviewer and virtual observations is fair to high with all agreement rates higher than 70%. This is consistent with previous literature comparing virtual and on-site observations (e.g., [10]). For observations where agreement is high (e.g., type of dwelling unit, signs dwelling unit not well kept) or where virtual observations are more accurate than interviewer observations as compared with survey self-reports (such as presence of children), virtual observations conducted via GSV images can be an attractive alternative to replace interviewer observations as a source of auxiliary data. Replacing field interviewer observations with virtual observations could potentially save cost and effort that normally field interviewers would have to invest in order to collect observations in the field. In addition, with virtual observations, virtual observers can extend the area that they browse to a few blocks around the sample address to get a better observation of the neighborhood. This would save operational cost compared when field interviewers are asked to travel a few blocks around the sample address.

Further work is needed to develop the observational items and training virtual observers, especially on items that are subjective in nature. While agreement rates were relatively high, the reliability of the measures were quite low. The discrepancy between these is due to the difficulty of coding characteristics that are relatively uncommon (e.g., dangerous neighborhood). Virtual observers generally agreed when the characteristic was clearly present, but were not as consistent when it was not straightforward. This points to both improving procedures for the virtual observers and the field interviewers.

We examined the predictive power of virtual observations using GSV images in predicting response status and crime incidence aggregated at the household level. We found that virtual observations have comparable explanatory power as field interviewer observations in explaining variation in likelihood to respond to the screener interview and to report crime victimization. Furthermore, virtual observations of dwelling unit type are marginally significantly related with sample address’s likelihood to respond to the screener interview and significantly related with sample address’s likelihood to report any crime victimization, making it an ideal candidate for nonresponse adjustment.

Another advantage of virtual observations through GSV images is that virtual observations are conducted in a controlled environment, and thus, are less prone to environmental or contextual factors that would otherwise affect interviewers’ observations in the field. Furthermore, monitoring of virtual observers conducting observations through GSV images is easier to implement than monitoring of field interviewers conducting the work on site. The performance of virtual observers will be improved with close monitoring by in-house supervisors. Recent developments in GSV methodology [26] have the potential to assist and improve performance of virtual observers.

A major limitation of the study is that the findings of this study are based on a small sample of addresses in a crime study, which restricted the power to detect small differences and the ability to extend our results to other surveys. We suggest that future research replicate our methodology and evaluate other types of virtual observations.

## Conclusions

Our findings of this small-scale feasibility study on the use of virtual observations via Google Street View are promising, providing evidence that Google Street View can be used to produce a new type of paradata that can be used for adaptive survey designs and for nonresponse adjustment.

## Supporting information

S1 Data (XLSX)

## References

[1] Kreuter F. Improving Surveys with Paradata: Analytic Uses of Process Information. Wiley. 2013.

[2] Sinibaldi J, Trappmann M, Kreuter F. Which is the better investment for nonresponse adjustment: Purchasing commercial auxiliary data or collecting interviewer observations? Public Opinion Quarterly. 2014; 78:440–473.

[3] Kirgis N, Lepkowski J. A management model for continuous data collection: Reflections from the National Survey of Family Growth, 2006–2010. NSFG Paper (10–011). 2010. September 20, 2019, from the Population Studies Center website: http://citeseerx.ist.psu.edu/viewdoc/download?doi=10.1.1.642.2725&rep=rep1&type=pdf

[4] Schouten B, Shlomo N, Skinner C. Indicators for monitoring and improving representativeness of response. Journal of Official Statistics. 2011; 27:1–24.

[5] Groves RM, Heeringa SG. Responsive design for household surveys: tools for actively controlling survey errors and costs. Journal of the Royal Statistical Society: Series A (Statistics in Society), 2006;169:439–457.

[6] Olson K. Paradata for nonresponse adjustment. The ANNALS of the American Academy of Political and Social Science. 2013; 645: 142–170.

[7] West BT, Kreuter F. Factors affecting the accuracy of interviewer observations: Evidence from the National Survey of Family Growth. Public Opinion Quarterly. 2013; 77: 522–548.

[8] Walsh R, Dahlhamer J, Bates N. Assessing interviewer observations in the NHIS. Paper presented at the Joint Statistical Meting. 2013.

[9] Cornesse C. The utility of auxiliary data for survey response modeling: Evidence from the German Internet Panel. Survey Methods: Insights from the Field, Special issue: ‘Fieldwork Monitoring Strategies for Interviewer-Administered Surveys’. 2020. https://surveyinsights.org/?p=11849.

[10] Ren W, Krenzke T, West BT, Cantor D. An evaluation of the quality of interviewer and virtual observations and their value for nonresponse bias reduction. Survey Research Methods. 2022; 16:97–131.

[11] Matsuoka R, Maeda T. Neighborhood and individual factors associated with survey response behavior: A multilevel multinomial regression analysis of a nationwide survey in Japan. Social Science Japan Journal. 2015; 18: 217–232.

[12] Jacoby A, Lobo AP, Salvo JJ. Projecting local survey response in a changing demographic landscape: A case study of the Census in New York city. Journal of Survey Statistics and Methodology. 2022; 10:203–224.

[13] De Nadai M, Xu Y, Letouzé E, González MC, Lepri B. Socio-economic, built environment, and mobility conditions associated with crime: A study of multiple cities. Scientific Reports. 2020; 10:1–12. 32807802 10.1038/s41598-020-70808-2 PMC7431538

[14] Little R, Varivarian S. Does weighting for nonresponse increase the variance of survey means? Survey Methodology. 2005; 31:161–168.

[15] Matsuo H, Billiet J, Loosveldt G. Response-based quality assessment of ESS Round 4: Results for 24 countries based on contact files. 2010. September 20, 2019, from the European Social Survey website: https://www.europeansocialsurvey.org/docs/round4/methods/ESS4_response_based_quality_assessment_e02.pdf.

[16] Hidiroglou M, You Y. Comparison of unit level and areal level small area estimators. Survey Methodology. 2016;42:41–61.

[17] Kreuter F, Olson K, Wagner J, Yan T, Ezzati-Rice TM, Casas-Cordero C, et al. Using proxy measures and other correlates of survey outcomes to adjust for non-response: examples from multiple surveys. Journal of the Royal Statistical Society: Series A (Statistics in Society). 2010;173:389–407.

[18] West BT, Kreuter F, Trappmann M. Is the collection of interviewer observations worthwhile in an economic panel survey? New evidence from the German Labor Market and Social Security (PASS) study. Journal of Survey Statistics and Methodology. 2014; 2:159–181.

[19] Uribe-Toril J, Ruiz-Real JL, Galindo AC. et al. How to use Google street view for a time-lapse data collection methodology: potential uses for retailing. Journal of Ambient Intelligence and Humanized Computing. 2023; 14:2199–2209.

[20] Li X, Zhang C, Li W. Building block level urban land-use information retrieval based on Google Street View images. GIScience & Remote Sensing. 2017;54:819–835.

[21] Nesse K, Airt L. Google Street View as a replacement for in-person street surveys: Meta-analysis of findings from evaluations. Journal of Urban Planning and Development. 2020;146. doi: 10.1061/(ASCE)UP.1943-5444.0000560

[22] Clarke P, Ailshire J, Melendez R, Bader M, Morenoff J. Using Google Earth to conduct a neighborhood audit: reliability of a virtual audit instrument. Health Place. 2010 Nov;16(6):1224–9. doi: 10.1016/j.healthplace.2010.08.007 20797897 PMC2952684

[23] Griew P, Hillsdon M, Foster C. et al. Developing and testing a street audit tool using Google Street View to measure environmental supportiveness for physical activity. Int J Behav Nutr Phys Act. 2013; 10:103. doi: 10.1186/1479-5868-10-103 23972205 PMC3765385

[24] Phillips CB, Engelberg JK, Geremia CM, et al. Online versus in-person comparison of Microscale Audit of Pedestrian Streetscapes (MAPS) assessments: reliability of alternate methods. Int J Health Geogr 16, 27 (2017). doi: 10.1186/s12942-017-0101-0 28778205 PMC5545045

[25] Bader MDM, Mooney SJ, Bennett B, Rundle AG. The promise, practicalities, and perils of virtually auditing neighborhoods using Google Street View. The ANNALS of the American Academy of Political and Social Science. 2017;669:18–40.

[26] Uribe-Toril J, Galindo AC, Torres JA, De Pablo J, Ruiz-Real JL. Local development and gentrification resulting from the rehabilitation of singular buildings: Analysis of neural networks. Remote Sensors. 2021;13:1500.

[27] Rzotkiewicz A, Pearson AL, Dougherty BV, Shortridge A, Wilson N. Systematic review of the use of Google Street View in health research: Major themes, strengths, weaknesses and possibilities for future research, Health & Place. 2018;52:240–246. doi: 10.1016/j.healthplace.2018.07.001 30015181

[28] Vercruyssen A, Loosveldt G. Using Google Street View to validate interviewer observations and predict nonresponse: A Belgian case study. Survey Research Methods. 2017;11: 345–360.

[29] Wu Y, Nash P, Barnes LE, Minett T, Matthews FE, Jones A, et al. Assessing environmental features related to mental health: a reliability study of visual streetscape images. BMC Public Health. 2014;14:1094. doi: 10.1186/1471-2458-14-1094 25335922 PMC4219017

[30] Odgers CL, Caspi A, Bates CJ, Sampson RJ, Moffitt TE. Systematic social observation of children’s neighborhoods using Google Street View: a reliable and cost-effective method. J. Child Psychol. Psychiatry. 2012;53:1009–1017. doi: 10.1111/j.1469-7610.2012.02565.x 22676812 PMC3537178

[31] Charreire H, Mackenbach JD, Ouasti M, Lakerveld J, Compernolle S, Ben-Rebah M, et al. Using remote sensing to define environmental characteristics related to physical activity and dietary behaviours: a systematic review (the SPOTLIGHT project). Health Place. 2014;25:1–9. doi: 10.1016/j.healthplace.2013.09.017 24211730

[32] McHugh ML. Interrater reliability: the kappa statistic. Biochem Med. 2012; 22:276–82. doi: 10.1016/j.jocd.2012.03.005 23092060 PMC3900052

[33] Cicchetti DV, Feinstein AR. High agreement but low kappa: II. Resolving the paradoxes. Journal of clinical epidemiology. 1990;43:551–558. doi: 10.1016/0895-4356(90)90159-m 2189948

[34] Feinstein AR, Cicchetti DV. High agreement but low kappa: I. The problems of two paradoxes. Journal of clinical epidemiology. 1990;43:543–549. doi: 10.1016/0895-4356(90)90158-l 2348207

